# Microbiological and Clinical Assessments of Suture Materials and Cyanoacrylate Application in Impacted Third Molar Surgeries: A Scoping Review

**DOI:** 10.3390/jfb14100529

**Published:** 2023-10-20

**Authors:** Andrea Scribante, Martina Ghizzoni, Matteo Pellegrini, Pier Paolo Poli, Carlo Maiorana, Francesco Spadari

**Affiliations:** 1 Section of Dentistry, Department of Clinical, Surgical, Diagnostic and Pediatric Sciences, University of Pavia, 27100 Pavia, Italy; martina.ghizzoni01@universitadipavia.it; 2 Maxillofacial Surgery and Dental Unit, Fondazione IRCCS Cà Granda Ospedale Maggiore Policlinico, 20122 Milan, Italy; pierpaolo.poli@unimi.it (P.P.P.); carlo.maiorana@unimi.it (C.M.); francesco.spadari@unimi.it (F.S.); 3 Department of Biomedical, Surgical and Dental Sciences, University of Milan, Via della Commenda 10, 20122 Milan, Italy

**Keywords:** cyanoacrylate, dentistry, impacted third molar, oral surgery, PTFE, silk suture, Vicryl

## Abstract

The extraction of impacted third molars is a common but potentially complication-prone oral surgical procedure. Wound healing plays a vital role in preventing complications. This scoping review aimed to assess the clinical and microbiological aspects of various suture materials and cyanoacrylates. Unlike existing studies, we included more articles and comprehensively compared suture materials. Articles published in languages other than English; duplicate studies; studies deemed irrelevant for the specific research questions, including those analyzing different supplementary treatments or not corresponding to the abstract’s content; ex vivo or experimental animal studies; studies lacking approval from an ethics committee; and narrative reviews, systematic reviews, or systematic and meta-analysis reviews were excluded. Thus, only 17 studies, published between 2000 and 2023, were included in the search. Suture techniques varied among surgeons, with debates on primary and secondary closure methods. A comparison of different suture materials and their effects on wound healing, infection rates, and other factors was described. Cyanoacrylate has also been used as an alternative to traditional sutures. Microbiological analysis showed varying bacterial adhesion based on the suture material, with silk sutures retaining more microbes than PTFE sutures. Clinical assessments have revealed differing inflammatory responses that affect wound healing and complications. Cyanoacrylate has emerged as a promising alternative to traditional sutures, owing to its rapid polymerization and early healing. However, the choice of suture material in impacted third molar surgery remains controversial, considering microbiological factors and clinical outcomes. More extensive randomized clinical trials are required to better understand the effect of suture materials on surgical outcomes and potential improvements. This study could enhance the safety and effectiveness of this common oral surgical procedure.

## 1. Introduction

Extraction of the third molars represents the prevailing and highly requested surgical treatment within the field of oral and maxillofacial surgery [1]. Third molars manifest the highest prevalence of impaction and harbor the capacity for incomplete eruption into a normal functional disposition. The incidence of impacted third molars ranges from 16.7% to 68.6% among heterogeneous demographic cohorts [2,3,4,5,6,7,8,9,10].

Surgical procedures are generally uneventful but can lead to complications, with reported rates in impacted third molar surgeries ranging from 4.6% to 30.9% [11,12]. These complications include intraoperative issues like bleeding, damage to adjacent teeth, and fractures, as well as postoperative problems like swelling, pain, and infection [13,14]. Effective wound closure is crucial to prevent these complications [15].

Sutures come in two categories: absorbable (e.g., polyglactin) and nonabsorbable (e.g., silk and PTFE), with nonabsorbable sutures being commonly used [15,16]. Synthetic sutures are preferred for their reliability, manageability, and minimal tissue reactions [17]. Multifilament sutures offer strength and flexibility, while monofilament sutures have less resistance but need special attention during handling [17,18]. Ideal suture materials should resist pulling forces, remain stable, and be biologically inert to reduce infection risk [19,20,21,22,23].

Cyanoacrylate is gaining popularity as a tissue adhesive in lower third molar extractions due to its rapid polymerization, tissue adherence, and natural detachment [15,24]. However, it has limitations like handling difficulties, a learning curve, and higher cost, and there have been reports of potential allergic reactions and thrombotic events when used intravascularly [25].

Despite the existence of numerous relevant studies in the recent literature, there is a lack of comprehensive reviews that deeply analyze the clinical and microbiological aspects of different suture materials and their comparison with cyanoacrylate in impacted third molar surgeries. This study aims to address this gap by conducting a scoping review that specifically evaluates the existing literature on suture materials and cyanoacrylate used in these surgeries.

To provide a clear framework for our review, it is important to note that our focus is exclusively on randomized controlled trials and observational cohort studies. This choice is driven by our intention to rely on the most robust and clinically relevant evidence. We exclude in vitro studies, as our primary interest lies in the clinical and microbiological outcomes associated with these materials in human surgical settings.

Our objective is to offer a comprehensive overview of the available research, emphasizing both the clinical and microbiological aspects of different suture materials and cyanoacrylate applications in impacted third molar surgeries. By doing so, we aim to provide valuable insights for practitioners and researchers in the field of oral and maxillofacial surgery.

## 2. Materials and Methods

### 2.1. Focused Questions

What are the microbiological and clinical features of the various suture materials currently used in impacted third molar surgeries? Is cyanoacrylate a better alternative to traditional sutures?

### 2.2. Eligibility Criteria

The inclusion criteria considered for this review were (I) study design—interventional studies, observational studies; (II) patients undergoing impacted third molar surgeries; (III) interventions—wound suture/closure after third molar surgeries; and (IV) outcome—clinical results after wound suturing/closure. The analysis was limited to studies that satisfied all the inclusion criteria, while the exclusion criteria comprised the following aspects: (I) abstracts of articles published in languages other than English; (II) duplicate studies; (III) studies deemed irrelevant to the specific research questions, including those analyzing different supplementary treatments or not corresponding to the abstract’s content; (IV) ex vivo or experimental animal studies; (V) studies without ethics committee approval; and (VI) narrative, systematic, or meta-analysis reviews.

### 2.3. Search Strategy

A three-stage search process was executed following the methodology described by the Joanna Briggs Institute (JBI) for scoping reviews. Initially, preliminary and restricted exploration was carried out using PubMed (MEDLINE) and Scopus. Subsequently, the relevant terminology was extracted from the articles to formulate an all-encompassing research strategy. Finally, the reference lists of all articles were searched to identify any additional pertinent research [26].

Furthermore, the application of the population–concept–context (PCC) framework was incorporated. This framework is grounded in three fundamental aspects: population (individuals undergoing third molar surgeries), concept (varied suture materials for wound closure), and context (without confinement to any specific cultural or environmental component). Scrutiny of study abstracts investigating the different suture materials used during third molar surgeries was conducted. Throughout this comprehensive literature review, adherence was maintained to the preferred reporting items for scoping reviews (PRISMA-ScR) consensus, as depicted in Table S1 [27].

### 2.4. Research

The terms used for Medical Subject Headings (MeSH) included “tooth impacted”, “molar third”, “polyglactin 910”, “silk” “polytetrafluoroethylene”, and “cyanoacrylates”. Electronic exploration was performed using the PubMed (MEDLINE) and Scopus databases. Articles published between 2000 and 2023 were included. Data were extracted between June 2023 and September 2023, and a final search was conducted on 16 September 2023.

The search was conducted by two reviewers (M.G. and M.P.). Any disparities that emerged during the review were resolved by consensus. For complex cases, four additional reviewers (A.S., P.P.P., C.M., and F.S.) were consulted. The initial phase of screening involved the assessment of article titles and abstracts, excluding irrelevant studies. Subsequently, the relevant articles underwent a comprehensive evaluation involving a thorough examination of their full content. The outcomes were carefully recorded, and similar studies that met the predetermined inclusion criteria were identified and incorporated in this review.

The present protocol was registered on the Open Science Framework platform (Registration DOI https://doi.org/10.17605/OSF.IO/DNJHS, accessed on 16 September 2023).

The strategies applied to each electronic database are listed in Table S2.

### 2.5. Quality Assessment of Included Studies

In this study, the potential for bias in clinical studies was appraised through a qualitative analysis using the National Heart, Lung, and Blood Institute (NHLBI) (Bethesda, MD, USA) Quality Assessment of Controlled Intervention Studies framework for Observational Cohort and Cross-Sectional Studies. This approach enabled a comprehensive and methodical evaluation of the quality and potential biases within the included studies, aiming to establish the dependability and credibility of the results [28].

## 3. Results

**Figure 1:**
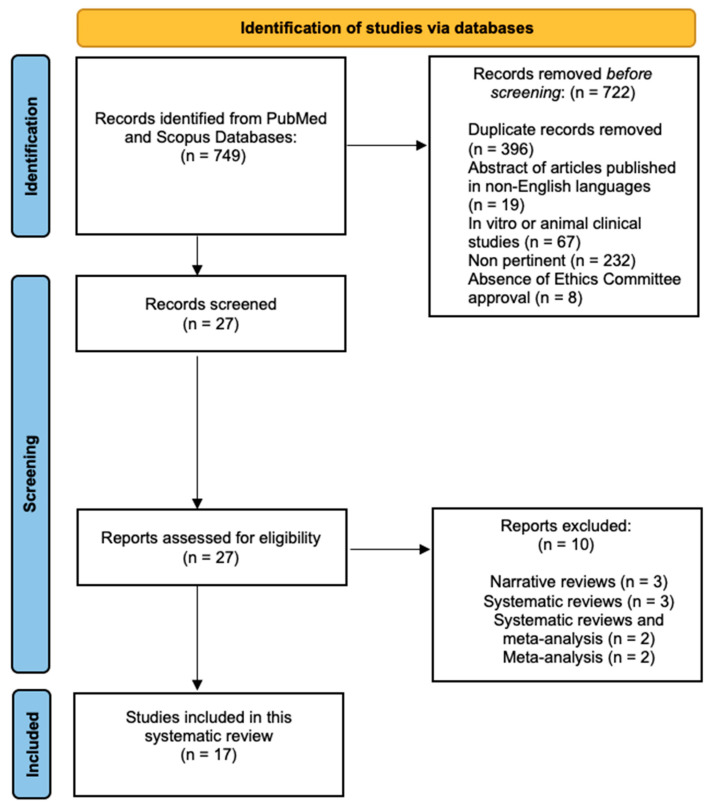
Flowchart of the review process.

The initial search using Medical Subject Headings (MeSH) terms resulted in 749 articles. A total of 722 articles were excluded for various reasons: 19 abstracts were in languages other than English, 396 were duplicates, 67 were related to in vitro or animal clinical studies, 232 were not relevant to the research topic, and 8 were not approved by an ethics committee. Following this initial screening, 27 articles underwent further assessment based on their titles and abstracts. Among these, 27 full-text articles fulfilled the eligibility criteria and were included in the in-depth analysis. Simultaneously, 10 full-text articles were excluded because they were narrative reviews, systematic reviews, or meta-analyses. Ultimately, a total of 17 pertinent articles were comprehensively reviewed and scrutinized as part of this examination. Figure 1 shows a flowchart of the review procedure.

Table S3 displays the research papers not considered in this analysis and the explanations for their exclusion [29,30,31,32,33,34,35,36,37,38].

The studies were controlled intervention [39,40,41,42,43,44,45,46,47,48,49,50,51,52], observational cohort, and cross-sectional studies [22,53,54].

### Risk of Bias

The assessment of bias risk in the articles included in this review was conducted using the Cochrane Collaboration tool. The criteria used to evaluate the risk of bias are outlined in Table S4. The outcomes of this assessment are shown in Table 1, revealing a moderate level of risk of bias.

Table 2 presents the baseline characteristics of patients included in the selected studies. A detailed overview of the evidence obtained from the studies included in this review is presented in Table S5 (Supplementary Materials). This information included the study design and objectives, research methods used, findings, and conclusions drawn by the authors of each study. The NHLBI Quality Assessment Tool for Controlled Intervention Studies is presented in Table S6 (Supplementary Materials). Similarly, Table S7 (Supplementary Materials) displays the NHLBI Quality Assessment Tool for Observational Cohort and Cross-Sectional Studies.

## 4. Discussion

Seventeen studies fitting into two categories (controlled intervention and observational/cohort studies) were included in this scoping review.

The selection of a suture technique following third molar surgery is still debated among surgeons and in published articles. Primary and secondary intention healing presents a complex challenge for many surgeons as they aim to enhance recovery and achieve improved surgical outcomes. While many surgeons prefer primary closure after impacted third molar surgery to enhance blood clot adhesion, minimize food impaction, and prevent alveolar osteitis, secondary closure appears more proficient in alleviating postoperative discomfort, minimizing facial swelling, and reducing trismus [46]. Moreover, there is no consensus concerning the appropriate role of sutures and the potential advantages of sutureless techniques following impacted third molar surgery, with conflicting findings [55,56].

### 4.1. Microbiological Aspects

Sutures employed following third molar surgery can act as a surface for bacterial adhesion, leading to an inflammatory response [57]. This phenomenon can be attributed to needle-induced trauma, the presence of stitches within the socket, or the buildup of bacterial plaque on the stitching wire [58].

After one week, the microbiological analysis of the removed sutures reveals significantly higher microbial retention on silk sutures compared to PTFE sutures, regardless of the culture medium used [53,59].

In contrast, some studies showed that silk sutures exhibit the least attraction for bacteria, while other suture materials show significant bacterial proliferation. Particularly, nonabsorbable multifilament sutures coated with substances such as polyethylene vinyl acetate, polybutylene, or silicone display the greatest rates of microbial growth [41,60]. This may be due to different study designs, methodologies, or specific conditions under which the experiments were conducted.

Various aerobic bacterial strains, including Streptococcus species (such as *S. mitis, S. sanguis*, *S. oralis*, *S. mutans*, and *Gemella morbillorum*, *Staphylococcus warneri*, *Neisseria* species, *Actinomyces* species, and *Pasteurella* species) as well as anaerobic bacterial strains like *Veillonella parvula*, *Peptostreptococcus* species, *Actinobacillus* species, *Prevotella* species, and *Fusobacterium* species, are predominantly present on all varieties of stitching materials. Nonresorbable sutures harbor more bacteria than absorbable ones, with the total count of facultative anaerobic bacteria isolated nearly twice as high [22,41].

Clinical observation reveals a dense layer of dental plaque coated on the entire surface of the polyester sutures after 1 week. The only sutures free from the accumulation of dental plaque are the ones made of polypropylene [54].

Anaerobic bacterial species, which are more pathogenic than their aerobic counterparts, tend to accumulate in plaque deposits at a higher concentration [46].

Vicryl Plus sutures, which are coated with triclosan, can lead to a significant reduction in both the ratio of CFU (colony-forming units), length to the total number of bacterial colonies, and the CFU–length ratio of *Lactobacillus* colonies compared to using Vicryl sutures. While it also led to a decrease in the CFU–length ratio of *S. mutans* colonies, this reduction was not statistically significant in the patients analyzed [39]. These microorganisms are known to be significant contributors to surgical site infections in the skin [61].

Regarding microbial adhesion, the gold standard in clinical practice involves using synthetic sutures to minimize it [54,62].

### 4.2. Clinical Evaluation

The immediate post-surgical period is often challenging to endure, as it is when most complications tend to occur. Inflammatory tissue responses to suture materials, infection, and wound dehiscence are common concerns during this time [63].

The literature showed that the highest level of inflammatory response in the observation period—up to 3 postoperative weeks—was with polyglycolic acid, then with catgut, and the lowest with Vicryl Rapide. The occurrence of local reaction at 1 week post-surgery presented a statistically significant distinction when comparing catgut with Vicryl Rapide, as well as polyglycolic acid with Vicryl Rapide [47,64].

Furthermore, it has been verified that Vicryl Rapide promotes accelerated wound healing in humans, resulting in reduced occurrences of dehiscence and milder local reactions when compared to the use of catgut or polyglycolic acid. A notable statistical contrast was observed in terms of dehiscence incidence after 72 h when comparing catgut and Vicryl Rapide, with Vicryl Rapide demonstrating a notably lower occurrence [47].

Enhanced healing was observed around polypropylene stitches compared to silk ones, both 72 h and 7 days following the impacted third molar surgery [42].

In addition to these signs, it is crucial to maintain minimal local reaction intensity (with a low antigenic potential) and minimize the risk of wound dehiscence as much as possible. Silk sutures from different brands showed the same outcomes being clinically comparable. There were no noteworthy differences in clinical indicators of inflammation and tissue responses, encompassing aspects such as swelling, discomfort, and restricted mouth opening in the extraction site. This held true for assessments made at both the 72-hour and 7-day post-surgery marks, as well as during all follow-up visits. Likewise, there were no significant differences observed in the rates of wound infection, suture loosening, total procedure duration, anesthetic dosage, intraoperative suture management variables, the time required for full wound healing and suture removal, or the occurrence of additional complications such as bleeding, alterations in taste, or other adverse events between the two suture groups [44].

Drug-coated sutures can enhance the clinical performance of suture materials; for instance, tadalafil/polycaprolactone improves wound healing processes by promoting vascular stimulation [65].

Poliglecaprone coated with triclosan, an antibacterial suture, was compared with silk sutures. Black silk exhibited significantly fewer bacteria (*p* < 0.001 at 72 h, *p* = 0.033 at 7th day); common ones included Gram-positive cocci, Gram-negative cocci, Gram-positive bacilli, and Gram-negative bacilli. Monocryl Plus, another antibacterial suture, demonstrated its most potent antibacterial effect at 72 h, indicating potential benefits in controlling surgical site infections [43].

Polypropylene sutures have been shown to offer several advantages to clinicians due to minimal tissue drag, easy knot tying, and resilience to saliva and blood. Its properties greatly influence suture removal ease as well [40].

Synthetic monofilament sutures are the preferred choice in daily clinical practice for oral surgery procedures due to their ability to minimize tissue reactions [66].

### 4.3. Cyanoacrylate versus Suture

New materials testing is opening the scene to cyanoacrylate as a reliable alternative to classic sutures, especially in third molar surgeries. Cyanoacrylate (CA) glues are classified as synthetic hybrid tissue sealants. They are part of a chemical category known for their robust and rapidly acting adhesive characteristics, and they find widespread applications in industry, medicine, and everyday household tasks [67].

In the literature, there is debate about whether cyanoacrylate is better than classic sutures. If compared with silk suture, it emerges that there is no substantial variance in the degree of pain experienced between the two approaches. Cyanoacrylate shows less bleeding immediately post-surgery; however, after 72 h it is comparable with silk, with statistically significant results [52].

Considering pain and swelling, statistically, there was no significant difference found between silk and cyanoacrylate materials [45].

Cyanoacrylate tissue adhesive led to faster, less inflamed, and uniform early healing, likely due to its protective barrier isolating the wound from external influences, promoting consistent wound recovery [48]. Moreover, it exhibits significant efficacy as a bacteriostatic and hemostatic agent [51].

Comparing cyanoacrylate with Vicryl Rapide sutures after third molar surgery, cyanoacrylate showed significantly better immediate hemostasis (visual analog scale) but similar bleeding on days 2 and 7. Cyanoacrylate also had a shorter wound closure time (76.33 vs. 229.70 s). However, patients using cyanoacrylate needed more rescue analgesics. Despite tissue adhesive’s advantages, Vicryl Rapide sutures are favored for uncomplicated healing after third molar removal [49].

### 4.4. Study Limitations and Future Studies

This study has some limitations that should be addressed. The search process might have been too precise for a scoping question. Comparing results could be complex, and potentially influenced by the specific sample being analyzed. While all the included studies featured impacted third molars in a similar position, there were variations in depth, angulation, and classification. In addition, the establishment and evaluation of clinical parameters differed across studies.

Future studies, specifically more randomized clinical trials, employing robust protocols and substantial sample sizes, are required to delve deeper into the topic and develop new materials with better features. Finally, it would be interesting to examine the impact of other variables on the bacterial balance in the oral cavity in relation to the type of suture applied, such as probiotics [68], postbiotics [69], and other natural compounds [70].

## 5. Conclusions

This review discussed various aspects related to sutures used in impacted third molar surgery. It compared different suture materials and their effects on wound healing, infection rates, and other factors. Additionally, it introduced the application of cyanoacrylate as an innovative option to traditional sutures, highlighting the ongoing debate among surgeons. Key findings include the impact of suture materials on bacterial adhesion. Vicryl Plus sutures with triclosan reduce bacterial colonization. Different sutures lead to varying levels of inflammation and tissue reactions, with Vicryl Rapide showing faster healing. Polypropylene sutures offer advantages in ease of use. Cyanoacrylate adhesive is emerging as an alternative to traditional sutures with benefits in faster healing and potential hemostatic properties. Further research, particularly randomized clinical trials with robust protocols and augmented sample sizes, is needed to provide more comprehensive insights into the topic and potentially develop improved suture materials. 

## Figures and Tables

**Table 1:** Risk of bias of the studies included in this review: The green symbol represents a low risk of bias, whereas the yellow symbol represents a high risk of bias.

References (Authors, Year of Publication)	Random Sequence Generation	Allocation Concealment	Blinding	Incomplete Outcome Data	Selective Reporting
Etemadi et al., 2022 [39]	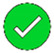	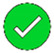	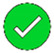	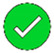	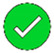
Dragovic et al., 2020 [40]	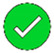	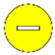	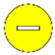	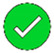	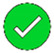
Banche et al., 2007 [41]	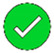	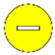	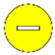	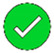	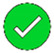
Dragovic et al., 2018 [42]	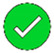	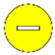	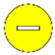	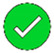	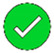
Sala-Perez et al., 2016 [43]	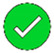	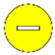	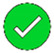	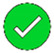	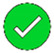
Balakrishna et al., 2022 [44]	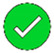	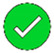	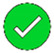	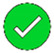	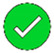
Oladega et al., 2019 [45]	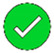	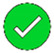	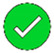	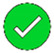	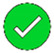
Bucci et al., 2017 [46]	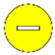	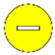	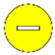	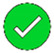	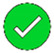
Gazivoda et al., 2015 [47]	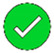	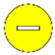	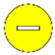	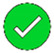	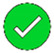
Thoniyottupurayil et al., 2022 [48]	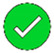	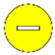	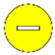	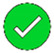	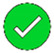
Pelia et al., 2021 [49]	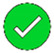	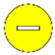	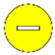	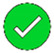	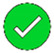
Joshi et al., 2011 [50]	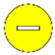	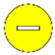	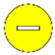	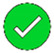	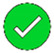
El-rewainy et al., 2015 [51]	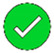	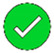	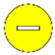	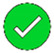	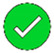
Ghoreishian et al., 2009 [52]	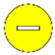	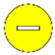	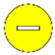	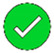	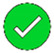
Parrini et al., 2023 [53]	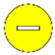	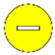	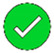	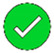	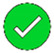
Yaman et al., 2022 [54]	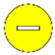	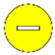	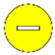	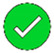	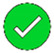
Otten et al., 2005 [22]	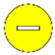	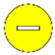	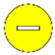	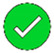	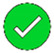

**Table 2:** Baseline characteristics of patients included in the selected studies.

References (Authors, Year of Publication, Origin of the Research, and Study Design)	N° of Patients and % Women Mean Age (Years), Mean (SD or Range)	Inclusion and Exclusion Criteria	Suture Materials
Etemadi et al., 2022 Iran RCT [39]	27 W: 77.78% 23.7 ± 3.1	Exclusion criteria: Systemic illnesses treated with systemic or oral medications that can disrupt the usual oral microbiota and colonization, tobacco and substance dependency, alcohol dependency, preexisting intraoral inflammation prior to the surgical procedure, pregnancy, breastfeeding, documented or suspected allergies to suture materials or other study-related materials, suture loss occurring within the first week, the presence of removable oral prosthetics, and instances of post-surgery infection or other scenarios warranting antibiotic treatment.	Vicryl; Vicryl Plus.
Dragovic et al., 2020 Serbia RCT [40]	32 W: 65.62% 18–25	Exclusion criteria: N.R.	Sofsilk^®^: nonabsorbable natural multifilament wax coated silk; Surgipro^®^: nonabsorbable synthetic monofilament polypropylene; Polysorb^®^: absorbable multifilament copolymer of glicolide and lactide 9:1 coated with Ca-stearate and *Ε*-caprolactone; Caprosyn^®^: absorbable monofilament copolymer of *E*-caprolactone, glicolide, trimethylene carbonate, lactide 6:2:2:1.
Banche et al., 2007 Italy RCT [41]	60 W: N.R. N.R.	Exclusion criteria: N.R.	Supramid (B. Braun, Melsungen, Germany): black, nonabsorbable, pseudomonofilament suture made of polyamide. Synthofil (B. Braun, Aesculap, Bethlehem, PA, USA): green, nonabsorbable, multifilament suture composed of braided polyethylene terephthalate fibers and coated uniformly with polyethylene vinyl acetate. Ethibond Excel (Johnson & Johnson Intl, Hamburg, Germany): green, nonabsorbable, braided suture composed of polyethylene terephthalate and coated with polybutylate. Monocryl (Johnson & Johnson Intl): violet, absorbable, monofilament suture prepared from a copolymer of glycolide and ε-caprolactone. Ti-Cron (Sherwood, Davis & Geck, Danbury, CT, USA): blue, nonabsorbable, braided multifilament suture composed of polyethylene terephthalate and coated with silicone.
Dragovic et al., 2018 Serbia RCT [42]	10 W: 100% 21–27	Exclusion criteria: N.R.	Sofsilk: black braided silk; Surgipro: polypropylene.
Sala-Perez et al., 2016 Spain RCT [43]	20 W: 50% 18–35 23.6 ± 4.77	Exclusion criteria: Individuals with systemic conditions (such as immune suppression, current infections, diabetes mellitus, or hematinic disorders), pregnant individuals, substance abusers, and patients exhibiting a considerable alcohol consumption.	Monocryl® Plus: poliglecaprone suture with triclosan; Silk suture.
Balakrishna et al., 2022 India RCT [44]	Group 1: 65 W: 66.15% Group 2: 64 W: 64.06% Group 1: 27.96 ± 5.75 Group 2: 27.87 ± 6.14	Exclusion criteria: individuals with pericoronitis or complicated impacted third molars, pregnant or lactating females, noncompliant patients. Silk-allergic subjects, patients with a medical history including systemic diseases such as diabetes mellitus, tuberculosis, hemostatic disorders, osteoporosis, or unstable/life-threatening conditions, or those currently undergoing radiation therapy were excluded. Participants taking any form of local or systemic medications, including aspirin, or undergoing anticoagulant therapy within 30 days before surgery, or with a history of substance abuse, were also excluded. Furthermore, individuals already enrolled in another research study or actively engaged in investigations conducted by the same investigator or center.	Trusilk®: natural nonabsorbable black braided sterile silk suture; Mersilk®: natural nonabsorbable black braided sterile silk suture.
Oladega et al., 2019 Nigeria RCT [45]	120 W: 62.5% 27.3 ± 6.9	Exclusion criteria: N.R.	Silk suture; Cyanoacrylate glue.
Bucci et al., 2017 Italy RCT [46]	30 W: N.R. 16–63	Exclusion criteria: N.R.	Silk; Nylon suture; Polyglycolic acid
Gazivoda et al., 2015 Serbia RCT [47]	96 W: N.R. N.R.	Exclusion criteria: N.R.	Catgut; Polyglycolic acid; Polyglactin 910.
Thoniyottupurayil et al., 2022 India RCT [48]	14 W: N.R. 18–35	Exclusion criteria: Individuals who were either pregnant or breastfeeding, those displaying symptoms of pericoronitis or active infections, individuals with harmful behaviors such as smoking and excessive alcohol consumption, and individuals with inadequate oral hygiene.	Silk suture; Cyanoacrylate.
Pelia et al., 2021 India Controlled study [49]	60 W: 55% 18–40	Exclusion criteria: Patients affected by systemic diseases, smokers.	Cyanoacrylate; Vicryl rapid.
Joshi et al., 2011 India Controlled study [50]	30 W: 63.33% 20–32	Exclusion criteria: N.R.	Silk suture; Cyanoacrylate.
El-rewainy et al., 2015 Egypt Controlled study [51]	20 W: 40% 18–30 24	Exclusion criteria: Individuals affected by systemic diseases, pregnant or breastfeeding. Patients with signs of pericoronitis or active infection. Smoking or addicted subjects and oral respirators.	Silk suture; Cyanoacrylate.
Ghoreishian et al., 2009 Iran Controlled study [52]	16 W: 56.25% 18–24	Exclusion criteria: N.R.	Silk suture; Cyanoacrylate.
Parrini et al., 2023 Italy Prospective study [53]	10 W: 30% 25–40 31	Exclusion criteria: Smoking and diabetes mellitus.	Silk; PTFE.
Yaman et al., 2022 Turkey Prospective study [54]	43 W: 74.41% N.R.	Exclusion criteria: Pregnant or lactating females; patients taking anticoagulant medications, patients affected by systemic viral, bacterial, or fungal infections; and smokers.	Poly (glycolide-co-lactide); Fast absorbable poly (glycolide-co-lactide); Poly-glycolic acid-cocaprolactone; Polydioxanone; Silk; Polypropylene; Polyvinylidene difluoride; Polyamide; Polyester; PTFE.
Otten et al., 2005 Germany Prospective study [22]	11 W: N.R. N.R.	Exclusion criteria: N.R.	Monocryl JB-1 70 cm 4/0 (Ethicon, Norderstedt, Germany) violet, monofilament, resorbable, copolymer of glycolide, and epsilon–caprolactone. Deknalon 45 cm 6-0 (Deknatel, Genzyme GmbH, Lubeck, Germany) blue, monofilament, nonresorbable, nylon.

Abbreviations: ASA: American Society of Anesthesiology; CFU: colony-forming units; N.R.: not Reported; PTFE: polytetrafluoroethylene; RCT: randomized controlled trial.

## Data Availability

Upon request to the corresponding author, the data are available for use. The protocol of the review was registered with the Open Science Framework (OSF) at https://doi.org/10.17605/OSF.IO/DNJHS and registered from osf.io/5sbny (accessed on 16 September 2023).

## Supplementary Materials

The following supporting information can be downloaded at: https://www.mdpi.com/article/10.3390/jfb14100529/s1, Table S1: PRISMA-ScR checklist; Table S2: Search strategies for electronic databases; Table S3: Summary table of studies excluded in this scoping review; Table S4: Criteria for judging risk of bias in the “Risk of bias” assessment tool; Table S5: Evidence of studies included in this scoping review; Table S6: NHLBI Quality Assessment Tool for Controlled Intervention Studies, Table S7: NHLBI Quality Assessment Tool for Observational Cohort and Cross-Sectional Studies.
