# Supplementary material for: Microbiological and Clinical Assessments of Suture Materials and Cyanoacrylate Application in Impacted Third Molar Surgeries: A Scoping Review
Source: J Funct Biomater. 2023 Oct 20;14(10):529. doi: 10.3390/jfb14100529 (PMC10607494; doi:10.3390/jfb14100529)
Supplement: Supplementary file 1 [file jfb-14-00529-s001.zip › jfb-2653646-supplementary.pdf]

Journal of
Functional Biomaterials

Supplementary Materials

Microbiological and Clinical Assessments of Suture Materials and Cyanoacrylate Application in Impacted Third Molar Surgeries: A Scoping Review

Andrea Scribante [1,*], Martina Ghizzoni [1], Matteo Pellegrini [2,3,*], Pier Paolo Poli [2,3], Carlo Maiorana [2,3] and Francesco Spadari [2,3]

1 Section of Dentistry, Department of Clinical, Surgical, Diagnostic and Pediatric Sciences, University of Pavia, 27100 Pavia, Italy; martina.ghizzoni01@universitadipavia.it
2 Maxillofacial Surgery and Dental Unit, Fondazione IRCCS Cà Granda Ospedale Maggiore Policlinico, 20122 Milan, Italy; pierpaolo.poli@unimi.it (P.P.P.); carlo.maiorana@unimi.it (C.M.); francesco.spadari@unimi.it (F.S.)
3 Department of Biomedical, Surgical and Dental Sciences, University of Milan, Via della Commenda 10, 20122 Milan, Italy
* Correspondence: andrea.scribante@unipv.it (A.S.); matteo.pellegrini@unimi.it (M.P.)

Citation: Scribante, A.; Ghizzoni, M.; Pellegrini, M.; Poli, P.P.; Maiorana, C.; Spadari, F. Microbiological and Clinical Assessments of Suture Materials and Cyanoacrylate Application in Impacted Third Molar Surgeries: A Scoping Review. *J. Funct. Biomater.* **2023**, *14*, 529. https://doi.org/10.3390/jfb14100529

Academic Editors: Dileep Sharma, Poornima Ramamurthy, Kate Miller, Stephen Hamlet

Received: 22 September 2023
Revised: 10 October 2023
Accepted: 18 October 2023
Published: 20 October 2023

Copyright: © 2023 by the authors. Licensee MDPI, Basel, Switzerland. This article is an open access article distributed under the terms and conditions of the Creative Commons Attribution (CC BY) license (https://creativecommons.org/licenses/by/4.0/).

Table S1. PRISMA-ScR Checklist.

SECTION	ITEM	PRISMA-ScR CHECKLIST ITEM	REPORTED ON PAGE #
**TITLE**			
Title	1	Identify the report as a scoping review.	Title
**ABSTRACT**			
Structured summary	2	Provide a structured summary that includes (as applicable): background, objectives, eligibility criteria, sources of evidence, charting methods, results, and conclusions that relate to the review questions and objectives.	Abstract
**INTRODUCTION**			
Rationale	3	Describe the rationale for the review in the context of what is already known. Explain why the review questions/objectives lend themselves to a scoping review approach.	Initial introduction
Objectives	4	Provide an explicit statement of the questions and objectives being addressed with reference to their key elements (e.g., population or participants, concepts, and context) or other relevant key elements used to conceptualize the review questions and/or objectives.	End of introduction
**METHODS**			
Protocol and registration	5	Indicate whether a review protocol exists; state if and where it can be accessed (e.g., a Web address); and if available, provide registration information, including the registration number.	Dedicated section in M&M
Eligibility criteria	6	Specify characteristics of the sources of evidence used as eligibility criteria (e.g., years considered, language, and publication status), and provide a rationale.	Dedicated section in M&M
Information sources*	7	Describe all information sources in the search (e.g., databases with dates of coverage and contact with authors to identify additional sources), as well as the date the most recent search was executed.	Dedicated section in M&M
Search	8	Present the full electronic search strategy for at least 1 database, including any limits used, such that it could be repeated.	Dedicated section in M&M

SECTION	ITEM	PRISMA-ScR CHECKLIST ITEM	REPORTED ON PAGE #
Selection of sources of evidence†	9	State the process for selecting sources of evidence (i.e., screening and eligibility) included in the scoping review.	Dedicated section in M&M
Data charting process‡	10	Describe the methods of charting data from the included sources of evidence (e.g., calibrated forms or forms that have been tested by the team before their use, and whether data charting was done independently or in duplicate) and any processes for obtaining and confirming data from investigators.	Dedicated section in M&M
Data items	11	List and define all variables for which data were sought and any assumptions and simplifications made.	Dedicated section in M&M
Critical appraisal of individual sources of evidence§	12	If done, provide a rationale for conducting a critical appraisal of included sources of evidence; describe the methods used and how this information was used in any data synthesis (if appropriate).	Dedicated section in M&M
Synthesis of results	13	Describe the methods of handling and summarizing the data that were charted.	Dedicated section in M&M
**RESULTS**			
Selection of sources of evidence	14	Give numbers of sources of evidence screened, assessed for eligibility, and included in the review, with reasons for exclusions at each stage, ideally using a flow diagram.	Dedicated table
Characteristics of sources of evidence	15	For each source of evidence, present characteristics for which data were charted and provide the citations.	Dedicated table
Critical appraisal within sources of evidence	16	If done, present data on critical appraisal of included sources of evidence (see item 12).	Dedicated table
Results of individual sources of evidence	17	For each included source of evidence, present the relevant data that were charted that relate to the review questions and objectives.	Dedicated table

SECTION	ITEM	PRISMA-ScR CHECKLIST ITEM	REPORTED ON PAGE #
Synthesis of results	18	Summarize and/or present the charting results as they relate to the review questions and objectives.	Dedicated table
**DISCUSSION**			
Summary of evidence	19	Summarize the main results (including an overview of concepts, themes, and types of evidence available), link to the review questions and objectives, and consider the relevance to key groups.	Followed
Limitations	20	Discuss the limitations of the scoping review process.	Followed
Conclusions	21	Provide a general interpretation of the results with respect to the review questions and objectives, as well as potential implications and/or next steps.	Followed
**FUNDING**			
Funding	22	Describe sources of funding for the included sources of evidence, as well as sources of funding for the scoping review. Describe the role of the funders of the scoping review.	None

Table S2. Search strategies for electronic databases.

Database	Search strategy
PubMed (MEDLINE)	#1 "Tooth, Impacted" [MESH] OR (Impacted Tooth) OR (Teeth, Impacted) OR (Impacted Teeth)<br>#2 "Molar, Third" [MESH] OR (Molars, Third) OR (Third Molar) OR (Third Molars) OR (Tooth, Wisdom) OR (Wisdom Tooth) OR (Teeth, Wisdom) OR (Wisdom Teeth)<br>#3 "Sutures" [MESH] OR (Suture) OR (Staple, Surgical) OR (Staples, Surgical) OR (Surgical Staples) OR (Surgical Staple)<br>#4 "Polyglactin 910" [MESH] OR (Vicryl) OR (Poly(Lactide-Co-Glycoside)) OR (Dioxanedione Polymer with Dimethyldioxanedione Polymer) OR (Glycolic-Lactic Acid Polyester) OR (Polyglactin9 OR (Poly(Glycolide Lactide)Copolymer)<br>#5 "Silk" [MESH]<br>#6 "Polytetrafluoroethylene" [MESH] OR (Polytef) OR (PTFE) OR (TFE) OR (FEP) OR (Expanded PTFE) OR (Tarflen) OR (Fluoroplast) OR (GORE-TEX) OR (Teflon) OR (Fluon)<br>#7 "Cyanoacrylates" [MESH] OR (Cyanoacrylate)<br>#8 #1 OR #2 AND #3 AND #4<br>#9 #1 OR #2 AND #3 AND #6<br>#10 #2 AND #3 AND #5 AND #7

SCOPUS	#1 “Tooth, Impacted” [MESH] OR (Impacted Tooth) OR (Teeth, Impacted) OR (Impacted Teeth)<br>#2 “Molar, Third” [MESH] OR (Molars, Third) OR (Third Molar) OR (Third Molars) OR (Tooth, Wisdom) OR (Wisdom Tooth) OR (Teeth, Wisdom) OR (Wisdom Teeth)<br>#3 “Sutures” [MESH] OR (Suture) OR (Staple, Surgical) OR (Staples, Surgical) OR (Surgical Staples) OR (Surgical Staple)<br>#4 “Polyglactin 910” [MESH] OR (Vicryl) OR (Poly(Lactide-Co-Glycoside)) OR (Dioxanedione Polymer with Dimethyldioxanedione Polymer) OR (Glycolic-Lactic Acid Polyester) OR (Polyglactin9 OR (Poly(Glycolide Lactide)Copolymer)<br>#5 “Silk” [MESH]<br>#6 “Polytetrafluoroethylene” [MESH] OR (Polytef) OR (PTFE) OR (TFE) OR (FEP) OR (Expanded PTFE) OR (Tarflen) OR (Fluoroplast) OR (GORE-TEX) OR (Teflon) OR (Fluon)<br>#7 “Cyanoacrylates” [MESH] OR (Cyanoacrylate)<br>#8 #1 OR #2 AND #3 AND #4<br>#9 #1 OR #2 AND #3 AND #6<br>#10 #2 AND #3 AND #5 AND #7

Table S3. Summary table of studies excluded in this systematic review.

Excluded Studies	Exclusion Reasons
Santos et al., 2023 [1]	Systematic Review
Stran-Lo Giudice et al., 2023 [2]	Systematic Review
Petronis et al., 2020 [3]	Narrative Review
Ma et al., 2019 [4]	Meta-analysis
Bailey et al., 2020 [5]	Meta-analysis
Azab et al., 2022 [6]	Systematic review and meta-analysis
Zhang et al., 2021 [7]	Narrative review
Faris et al., 2022 [8]	Systematic review
Raut et al., 2022 [9]	Systematic review and meta-analysis
Borie et al., 2019 [10]	Narrative review

Table S4. Criteria for judging risk of bias in the "Risk of bias" assessment tool.

**Random Sequence Generation**	
Criteria for a judgement of 'Low risk' of bias.	The investigators describe a random component in the sequence generation process.
Criteria for the judgement of 'High risk' of bias.	The investigators describe a non-random component in the sequence generation process. Usually, the description would involve some systematic, non-random approach.<br>Other non-random approaches happen much less frequently than the systematic approaches mentioned above and tend to be obvious. They usually involve judgement or some method of non-random categorization of participants.
**Allocation Concealment**	
Criteria for a judgement of 'Low risk' of bias.	Participants and investigators enrolling participants could not foresee assignment because one of the following, or an equivalent method, was used to conceal allocation.
Criteria for the judgement of 'High risk' of bias.	Participants or investigators enrolling participants could possibly foresee assignments and thus introduce selection bias.
**Blinding**	
Criteria for a judgement of 'Low risk' of bias.	Any one of the following:<br>- No blinding or incomplete blinding, but the review authors judge that the outcome is not likely to be influenced by lack of blinding;<br>- Blinding of participants and key study personnel ensured, and unlikely that the blinding could have been broken;<br>- No blinding of outcome assessment, but the review authors judge that the outcome measurement is not likely to be influenced by lack of blinding;<br>- Blinding of outcome assessment ensured, and unlikely that the blinding could have been broken.
Criteria for the judgement of 'High risk' of bias.	Any one of the following:<br>- No blinding or incomplete blinding, and the outcome is likely to be influenced by lack of blinding;

	- Blinding of key study participants and personnel attempted, but likely that the blinding could have been broken, and the outcome is likely to be influenced by lack of blinding;<br>- No blinding of outcome assessment, and the outcome measurement is likely to be influenced by lack of blinding;<br>- Blinding of outcome assessment, but likely that the blinding could have been broken, and the outcome measurement is likely to be influenced by lack of blinding.
**Incomplete Outcome Data**	
Criteria for a judgement of 'Low risk' of bias.	Any one of the following:<br>- No missing outcome data;<br>- Reasons for missing outcome data unlikely to be related to true outcome (for survival data, censoring unlikely to be introducing bias);<br>- Missing outcome data balanced in numbers across intervention groups, with similar reasons for missing data across groups;<br>- For dichotomous outcome data, the proportion of missing outcomes compared with observed event risk not enough to have a clinically relevant impact on the intervention effect estimate;<br>- For continuous outcome data, plausible effect size (difference in means or standardized difference in means) among missing outcomes not enough to have a clinically relevant impact on observed effect size;<br>- Missing data have been imputed using appropriate methods.
Criteria for the judgement of 'High risk' of bias.	Any one of the following:<br>- Reason for missing outcome data likely to be related to true outcome, with either imbalance in numbers or reasons for missing data across intervention groups;<br>- For dichotomous outcome data, the proportion of missing outcomes compared with observed event risk enough to induce clinically relevant bias in intervention effect estimate;

	- For continuous outcome data, plausible effect size (difference in means or standardized difference in means) among missing outcomes enough to induce clinically relevant bias in observed effect size;<br>- 'As-treated' analysis done with substantial departure of the intervention received from that assigned at randomization;<br>- Potentially inappropriate application of simple imputation.
**Selective Reporting**	
Criteria for a judgement of 'Low risk' of bias.	Any one of the following:<br>- The study protocol is available and all of the study's pre-specified (primary and secondary) outcomes that are of interest in the review have been reported in the pre-specified way;<br>- The study protocol is not available but it is clear that the published reports include all expected outcomes, including those that were pre-specified (convincing text of this nature may be uncommon).
Criteria for the judgement of 'High risk' of bias.	Any one of the following:<br>- Not all of the study's pre-specified primary outcomes have been reported;<br>- One or more primary outcomes is reported using measurements, analysis methods or subsets of the data (e.g., subscales) that were not pre-specified;<br>- One or more reported primary outcomes were not pre-specified (unless clear justification for their reporting is provided, such as an unexpected adverse effect);<br>- One or more outcomes of interest in the review are reported incompletely so that they cannot be entered in a meta-analysis;<br>- The study report fails to include results for a key outcome that would be expected to have been reported for such a study.

Table S5: Evidence of studies included in this review.

Authors and Year of Publication	Study Design and Aim	Methods	Results	Conclusions
Etemadi et al., 2022 **[11]**	A 3 months randomized clinical trial to o assess the success rate of an antibacterial suture, Vicryl Plus (Ethicon Inc, Johnson and Johnson Company, Somerville, NJ), in preventing bacterial growth in the surgical site of the mandibular third molar.	27 patients were included in this double-blinded randomized clinical trial study. Surgical extraction of the mandibular wisdom tooth was done, and the incision was managed by randomly using Vicryl Plus and Vicryl (Ethicon Inc) sutures. After 7 days, sutures were removed and assessed microbiologically. Indicator species of *Streptococcus mutans* and *Lactobacillus* were assessed, and the total number of colonies on each suture was counted.	There was a significant difference between the two suture materials in the colony number-length ratio of *Lactobacillus* ($P = 0.031$) and total bacterial colonies ($P = 0.016$), but not for *S. mutans* species ($P = 0.201$).	Antibacterial Vicryl suture can be useful to reduce bacterial accumulation on the suture material in third molar extraction surgery.
Dragovic et al., 2020 **[12]**	A 6-week randomized clinical trial to compare four different suture materials in terms of their influence on wound healing, microbial adherence, tissue reaction, and relevant clinical parameters which determine their clinical value.	Total number of 32 patients undergoing surgical extraction of four impacted third molars were involved in the study. Clinical parameters were estimated intraoperatively and during the control check-ups. Soft tissue healing around sutures were evaluated on the 3rd and 7th day postoperatively. Microbial colonization was assessed by means of qPCR. Also, histological analysis was done to assess inflammatory reaction.	Significantly better soft tissue healing was found around monofilament and synthetic sutures compared to multifilament and natural ones respectively. Soft tissue healing was significantly better around all sutures on the 7th day than on the 3rd day postoperatively.	Non-resorbable polypropylene suture showed superior clinical characteristics among all sutures. Moreover, the best healing of soft tissue and the least inflammatory reaction was found around this thread. The poorest soft tissue healing was found around non-resorbable silk suture. This suture elicited strongest inflammatory reaction and showed the greatest microbial adherence affinity compared to alternative sutures.

Banche et al., 2007 **[13]**	A 3 weeks RCT to compare microbial colonization on various intraoral suture materials from patients undergoing dental surgery.	During dentoalveolar surgery, various suture materials were used in 60 patients, who were randomly divided into 5 groups of 12. In each group, silk was placed intraorally in association with a different type of suture (ie, Supramid, Synthofil, Ethibond Excel, Ti-cron, Monocryl) at the same site to compare microbial colonization intraindividually. Eight days postoperatively, the sutures were removed, and adhered micro-organisms were isolated, counted, and identified through enzymatic activities and fermentation of sugars.	In all 60 patients, silk sutures exhibited the smallest affinity toward the adhesion of bacteria compared with considerable proliferation with nonresorbable multifilament sutures (Supramid, Syntho- fil, Ethibond Excel, Ti-cron). On the contrary, the microbial load was significantly lower when absorbable monofilament Monocryl was used. A greater quantity of bacteria was found on nonresorbable sutures than on absorbable ones, and nearly 2 times more facultative anaerobic bacteria were isolated in total.	Our results show that bacteria adhere with different affinity to various types of suture materials. Absorbable silk and Monocryl exhibited the smallest number of adherent bacteria. Colonization by pathogens on sutures leads to the recommendation that sutures should be removed as early as possible after surgery is performed, to eliminate or to limit the reservoir for oral pathogens. This recommendation is dependent on whether the suture is absorbable.
Dragovic et al., 2018 **[14]**	A 1-week RCT to compare polypropylene and silk suture ma- terials in terms of bacterial adherence and clinical features including the impact on soft tissue healing.	Ten healthy patients were included in this study. Unilateral upper and lower wisdom teeth were extracted at the same time and wounds were sutured with different threads (one monofilament – polypropylene – and one multifilament – silk suture). Stitches were removed seven days postoperatively. Real-time	Significantly more pronounced bacterial adherence was found on silk compared to polypropylene sutures ($p = 0.005$). *S*uperior intraoperative handling properties were registered suturing with polypro- pylene compared to silk (p =	Polypropylene suture material showed significantly lower bacterial adherence and superior clinical features compared to silk, including better soft tissue healing.

		polymerase chain reaction was used to analyze bacterial adherence. Intraoperative handling and ease of removal were assessed with the help of Visual Analogue Scale. Landry healing index was used for evaluation of soft tissue healing.	0.005). Soft tissue healing was significantly better around polypropylene sutures, both on the third and the seventh postoperative day (p = 0.016). Patient discomfort was slightly higher for polypropylene sutures, but without statistical significance.	
Sala-Perez et al., 2016 **[15]**	A 1-week split mouth RCT to evaluate the clinical and microbiological impact of an antibacterial suture (Monocryl® Plus) in the surgical removal of I3M.	The study was designed involving 20 patients programmed for the surgical removal of I3M. Each side was randomly sutured with Monocryl® Plus or silk suture and removed for microbiological study 72 hours and 7 days after surgery. Presence of SSI, wound bleeding and the degree of discomfort associated with each type of suture material (scored by means of a visual analog scale) were evaluated. The level of contamination of each material was observed under the scanning electron microscope.	Wound bleeding upon suture removing was slightly greater after 72 hours and 7 days with black silk suture, though the differences were not statistically significant (p=0.752 and p=0.113, respectively). Patient discomfort was very similar with both types of suture material (p=0.861). Only one case of SSI was recorded with black silk suture after 72 hours. Microbiologically, the antibacterial suture showed a lesser presence of microorganisms (p<0.001, at 72h and p=0.033 at 7th day, respectively). The most common bacterial species	The greatest antibacterial effect of Monocryl Plus suture was observed after 72 hours. According to most authors, there is no doubt that this antibacterial suture can provide little safety in the control of SSI.

			included grampositive cocci (*Streptococcus viridans* group, *Neisseria spp*., Coagulasenegative *Staphy lococcus* and *Peptostreptoco ccus*), gramnegative cocci (*Veillonella*), grampositive Bacilli (*Lactobacillus*), and gramnegative Bacilli (*Prevotella*).	
Balakrishna et al., 2022 **[16]**	A 14-months single blind, randomized study to compare tissue reaction/inflammation after 3 and 7 days of mucosal closure with Trusilk ® and Mersilk ® silk sutures, following impacted mandibular third molar removal.	This study included subjects (Trusilk ®, n=65 and Mersilk ®, n=64), requiring mucosal suturing following impacted mandibular third molar removal. The primary endpoint, incidence of pain, swelling and trismus at the extraction area on post-surgery day 3 and 7 was evaluated. The secondary endpoints, incidence of tissue reaction, wound infection, suture loosening, other complications, operative time, amount of anesthesia, intraoperative suture handling, time needed for complete wound healing and suture removal, and adverse events were also recorded.	Socio-demographic and intra-oral characteristics were comparable between the groups. In Trusilk ® and Mersilk ® groups, a gradually decreasing pain score, starting from day 0 post-surgery (42.17±22.38 vs. 45.97±22.20) to day 7 (8.40±11.93 vs. 8.28±12.13) to day 30 (1.98±0.89 vs. 1.75±0.76) was witnessed. After the surgery, 21.54% and 17.19% subjects in Trusilk ® and Mersilk ® groups, respectively, had no post-operative swelling, while at the last two visits none of the subjects had swelling. Non-significant difference in wound	The results indicated that the Trusilk ® and Mersilk ® silk sutures are clinically equivalent and can be used for mucosal closure after removal of an impacted mandibular third molar with a minimal rate of pain, swelling and trismus.

			infection, suture loosening, wound healing, bleeding, taste changes, operative time, amount of anesthesia, intraoperative suture handling, and time needed for complete wound healing and suture removal was noted among the groups. No suture-related adverse events were recorded.	
Oladega et al., 2019 **[17]**	A 1-week RCT to compare postoperative sequelae and wound healing outcome following closure of surgical wound with either cyanoacrylate tissue adhesive or silk suture.	Subjects with mesio-angularly impacted mandibular third molar were allocated randomly into 2 equal groups. The control group had wound closure with silk suture and study group with cyanoacrylate tissue adhesive. Subjects were followed up for 7 postoperative days. Postoperative pain, swelling, trismus, bleeding, wound dehiscence and wound infection were evaluated.	Sixty subjects in each group completed the study. No significant difference was observed in the mean postoperative pain, swelling, trismus, wound dehiscence and infection between the 2 groups. There was a statistically significant difference in postoperative bleeding between the 2 groups on postoperative day 1, with more bleeding in the control group.	This study shows that cyanoacrylate tissue adhesive compares favourably with silk suture as a wound closure material. In addition, cyanoacrylate tissue adhesive seems to have beneficial haemostatic effect on postoperative bleeding.
Bucci et al., 2017 **[18]**	A 1-week RCT to compare the bacterial colonization on different suture materials after a third molar extraction.	Thirty patients were randomly selected among people going under third molar extraction; they were divided into 3 groups	The amount of cocci and bacilli on the sutures analyzed shows that silk (Ethicon Silk) is the	A less plaque retention, and consequently a fewer bacterial presence, is crucial to minimize the inflammatory process and allow a

		and one suture type was used on each group. After 7 days distal stitches were removed by a single operator, placed in physiologic solution and analyzed after 2 or 3 hours. Patients followed the same postsurgical protocols; materials used were: Ethicon Silk® 4/0, B. Braun Dafilon® 4/0, and B. Braun Safil® 4/0.	higher level of retention material where monofilament (B. Braun Dafilon) is the lower. There is a difference between monofilament and polyglycolide (B. Braun Safil), as the former is less retentive than the latter, although not significantly so.	better tissue healing. Since the capability of brushing and, of course, the final personal hygiene depends on multiple variables, we must use surgical protocols able to minimize the effect of improper cleaning on the healing process: this statement implies the use of low plaque retention materials. The use of monofilament or polyglycolide threads in sutures can help reducing bacterial concentration and therefore promotes a faster and better healing.
Gazivoda et al., 2015 **[19]**	A 3-weeks RCT to examine the speed of wound healing and complications incidence, after the use of three different absorbable synthetic suture materials in oral surgery (catgut, Dexon and Vicryl rapide), and to ascertain which one is the most suitable for oral surgery.	The study was conducted on 96 patients undergoing root resection or surgical extraction of third molars. Each of the suture materials (catgut, Dexon and Vicryl rapide) was used for 8 root resections and 8 surgical third molar extractions in the maxilla, as well as in the mandible (a total of 32 surgical interventions for each suture material).	The faster wound healing was obtained with Vicryl rapide compared to other two suturing material tested. There was no significant difference regarding the presence of local reaction in all the three groups of patients on the 21st postoperative day.	The results of our clinical study point out that Vycril- rapid contributes more than catgut or Dexon to faster healing of human wounds, with fewer incidences of wound dehiscence and milder local reactions.
Thoniyottupurayil et al., 2022 **[20]**	A 1 week randomized controlled clinical trial to compare the efficacy of isoamyl 2-cyanoacrylat and 3–0 silk suture for the closure of wound after surgical removal of the impacted mandibular third molar.	fourteen patients of both sexes, with a range of 18–35 years of age. Patients were randomly assigned to one of two groups (study or control) each with a submerged mesioangular impacted mandibular third molar (Class II Position B of Pell and Gregory's classification).	Fourteen subjects were enrolled in this study. In cyanoacrylate treated wounds, there was a considerable clinical and statistical improvement. The time it took to close a wound using isoamyl 2-cyanoacrylate was shorter	The use of cyanoacrylate tissue adhesive is a good method for the closure of mucoperiosteal flaps that is capable of overcoming most of the complications associated with traditional silk sutures, as well as providing ease of manipulation, time savings, and safety.

		After the surgical extraction of an impacted tooth, the flaps were closed using isoamyl 2-cyanoacrylate (Mervilyte) tissue adhesive in the study group and using 3–0 silk sutures in the control group. Recorded the time taken for placement of silk suture or cyanoacrylate tissue adhesive for the closure of the surgical wound. Both patient groups were given similar medication and postoperative instruction. Patients were recalled on postoperative day 1, day 3, and day 7 for evaluating postoperative pain, bleeding, and wound healing.	(2.13 ± 0.61) whereas it was longer for the silk suture group (6.34 ± 1.86). Early hemostasis was achieved with isoamyl 2-cyanoacrylate. Postoperative discomfort and hemorrhage were reduced when compared to the silk suture group. In the isoamyl 2-cyanoacrylate group, wound healing was also improved.	
Pelia et al., 2021 **[21]**	A 2 years randomized controlled clinical trial to compare the efficacy of isoamyl 2-cyanoacrylate tissue adhesive with 3-0 vicryl rapide suture for wound closure after the removal of impacted mandibular third molar.	The sample consisted of 60 patients with unilateral impactions divided in two groups equally. In group I, wound closure was done with tissue adhesive and in group II with 3-0 vicryl rapide suture. Patients were evaluated for pain, swelling and trismus preoperatively, immediate postoperatively, postoperative day 2 and 7. Bleeding was assessed immediate postoperatively, postoperative day 2 and 7. Healing was	Statistically significant difference was observed in terms of pain on postoperative day 2 and 7 between both the groups (*p* value—0.028* and 0.002*). In the immediate postoperative period, there was statistically highly significant difference in bleeding between the two groups (*p* value—0.000**). Statistically significant difference was also observed in terms of	This study concluded that vicryl rapide suture is an optimal alternative for wound closure after removal of impacted mandibular third molar as compared to tissue adhesive.

		assessed on postoperative day 2 and 7.	wound healing on postoperative day 2 and 7 (*p* value—0.011* and 0.024* respectively). Statistically significant difference was observed for time of closure and total number of rescue analgesic taken between two groups (*p* value—0.000** and 0.000**, respectively).	
Joshi et al., 2011 **[22]**	A 3 weeks controlled study to compare the efficacy of cyanoacrylate (tissue glue) placement after surgical removal of impacted mandibular third molars.	Thirty patients with bilaterally impacted mandibular third molars were studied in this controlled clinical trial. One side closure after surgical removal of third molar was done with conventional sutures and other side with cyanoacrylate.	The data analysis showed that postoperative bleeding with cyanoacrylate method was less significant than with suturing on the first and second day after surgery. There was no significant difference in the severity of pain between the two methods.	This study suggested that the efficacy of both, cyanoacrylate and suturing in wound closure were similar in the severity of pain, but use of cyanoacrylate showed better hemostasis.
El-rewainy et al., 2015 **[23]**	A 1-week randomized controlled trial to evaluate the clinical post-operative complications after the use of N-butyl cyanoacrylate soft tissue adhesive in closure of mucoperiosteal flaps after the surgical extraction of impacted mandibular third molars	The study was conducted on twenty patients of both sexes ranging from 20 to 30 years of age.Patients were divided equally into two groups (Study and control groups) each with mesioangular impacted mandibular third molar (class II position B according to Pell and Gregory's classification). After	There was a statistically significant reduction of pain, bleeding, trismus, wound reaction on using the N-butyl cyanoacrylate (PeriAcryl 90) compared to sutures , concerning wound dehiscence and facial swelling , the	The use of the N-butyl cyanoacrylate (PeriAcryl 90) for the closure of mucoperiosteal flaps is a reliable method that can overcome most of complications faced on using conventional silk sutures in addition to ease of manipulation , time saving and safety factors.

	compared to the use of conventional silk sutures.	the surgical extraction of impacted teeth the flaps were closed using PeriAcryl 90 (Glustitch corporation, Delta, BC, Canada) soft tissue adhesive in the study group and using 3/0 silk sutures in the control group. Patients were evaluated for pain, bleeding, trismus, facial swelling, wound dehiscence and local reaction.	results of both materials were nearly the same.	
Ghoreishian et al., 2009 **[24]**	A 6-weeks, controlled study to evaluate and compare the efficacy of cyanoacrylate and suturing on postoperative pain and bleeding after impacted third molar surgery.	Sixteen patients with similar bone impaction and inclination of mandibular third molars on the right and left sides were studied in this controlled clinical trial. The third molar surgery was carried out in 2 stages, 4 weeks apart, under local anesthesia. After bone removal and tooth resection, the right flap was closed with 3-0 silk sutures and the left flap with cyanoacrylate. A visual analogical scale was used to evaluate the severity of pain and bleeding on postoperative days.	he data analysis showed that postoperative bleeding with cyanoacrylate method was less significant than with suturing on the first and second days after surgery (*P* ⊚ .05). There was no significant difference in the severity of pain between the 2 methods (*P* ⊚ .05).	This study suggested that the efficacies of cyanoacrylate and suturing in wound closure were similar in the severity of pain, but use of cyanoacrylate resulted in better hemostasis.
Parrini et al., 2023 **[25]**	A 4 months prospective study to assess the ability of two types of surgical sutures, Silk and polytetrafluoroethylene polymer (PTFE), to carry aerobic and anaerobic bacteria on wounds after mandibular third molar	This study sampled a total of 10 consecutive healthy patients for mandibular third molar surgery at the Oral Surgery School, Dentistry and Dental Prosthodontics, Department of Medical Biotechnologies,	All the patients attended the suture removal date, and all the sutures were present in the site. None of the surgical sites presented dehiscence. No stitch loss was reported,	The authors found the PTFE suture to be superior to the silk suture in a reduction in the bacterial biofilm in both aerobic and anaerobic evaluations after M3M surgery.

	surgery, with a collection of the stitches at the suture removal and study in the laboratory on the basis of colony-forming units.	University of Siena, Siena, Italy. The mean age of the patients was 31 years (range 25–40 years), seven patients were male and three patients were female. Inclusion criteria were: presence of a partially impacted mandibular third molar. Exclusion criteria were: smoking and diabetes mellitus. Extraction of the mandibular third molar was performed under local anesthesia: after the third molar surgery, two sutures were applied on the surgical site distally to the second mandibular molar: one single 3/0 silk stitch; one single 3/0 PTFE stitch. No sutures were applied on the release incision. Sutures were removed after 7 days and were immediately conserved and sent to the laboratory to be rated on the basis of colony-forming units (CFUs). CFUs were evaluated and reported on GraphPad Prism and transformed into its base 10 logarithm. Data were analyzed with a non-parametric Wilcoxon test, and p-values < 0.05 were evaluated as statistically significant.	and no patient reported mouth washing or tooth brushing in the surgery site. All interventions were uneventful and no major complications were reported after M3M surgery. Bacterial retention resulted as statistically greater in silk sutures rather than PTFE sutures, both in Brain Heart Infusion samples (p = 0.003) and Wilkins-Chalgren anaerobe samples (p = 0.002).	

Yaman et al., 2022 **[26]**	A 3-months prospective study to compare the effects of 10 different suture materials commonly used in dentoalveolar surgery on wound healing, their postoperative microbial colonization, and related clinical parameters.	A total of 172 suture samples from patients who had undergone extraction of impacted third molars were included in the study. The suture materials studied were poly-glycolide-colactide, fast absorbable poly-glycolide-colactide, poly-glycolic acid-cocaprolactone, polydioxanone, silk, polypropylene, polyvinylidene difluoride, polyamide, polyester, and polytetrafluoroethylene (PTFE). The microbial colonization in all sutures and clinical parameters were evaluated after 1 week.	Multifilament sutures had higher bacterial colonization compared with monofilament sutures ($P < .001$). No dental plaque accumulation was observed in any samples of polypropylene sutures. Polydioxanone, PTFE, and poly-glycolic acid-cocaprolactone sutures exhibited less postoperative slack compared with all other sutures after 1 week. Patients with silk, polyvinylidene difluoride, and PTFE sutures had less suture-related discomfort. According to the Landry index score, monofilament sutures demonstrated superior wound healing to multifilament sutures ($P = .019$). In addition, nonabsorbable sutures showed significantly better wound epithelization than absorbable sutures ($P < .001$).	Bacterial colonization and tissue reactions due to the surface properties of the suture affected the wound healing after dentoalveolar surgery. Multifilament sutures should not be applied for prolonged periods because of their tendency for microbial colonization. The tissue reaction to the absorbable suture materials may adversely affect wound healing.

Otten et al., 2005 **[27]**	A 3-week prospective study to compare bacterial colonization resorbable (Monocryl#) and nonresorbable (Deknalon#) monofilament sutures used in intraoral dentoalveolar surgery.	The sutures were applied in 11 patients during dental surgery. Eight days postoperative the sutures were removed, and the adhered bacteria were isolated and identified by biochemistry, morphology, antibiotic susceptibility, and gaschromatography. The colonization was studied by scanning electron microscopy. Aerobic and anaerobic bacteria were isolated in nearly equal colony-forming units (cfu) on each suture.	In comparison with Monocryl# about 15% more aerobic and anaerobic strains were isolated on Deknalon#. Regarding the pathogens only, about three times more anaerobic strains were isolated on both sutures in total. Additionally, more pathogens were found on Deknalon# than on Monocryl# (aerobic >40%, anaerobic >25%). The variety of bacteria correspond with purulent infections, not with normal oral flora. Intraindividual comparisons of cfu showed differences in dependence of the patient as described for subgingivale plaques. For the *in vitro* study the sutures were incubated with *Streptococcus intermedius* and *Prevotella intermedia* for 0.5 h. Scanning electron microscopy was performed to examine qualitatively the level of	More pathogens were found on Deknalon# than on Monocryl#. The colonization with pathogens on both sutures leads to the recommendation that the sutures should be removed as early as possible after the surgery. This recommendation is independent whether the suture is resorbable or not. The isolation of oral pathogens leads to the idea of adequate antimicrobial prophylaxis before suture removal.

bacterial adherence. After 0.5 h the bacteria adhered very well. The colonization rate of *Streptococcus intermedius* on both sutures was similar. Coccoid bacteria within biofilms were seen. The growth of *Prevotella intermedia* was much better on Deknalon# than on Monocryl#. The risk of bacteremia at the time of suture removal is discussed.

Abbreviations: CFUs: Colony-Forming Units, I3M: Impacted Third Molar, PTFE: Polytetrafluoroethylene, CR: Quantitative Polymerase Chain Reaction RCT: Randomized Clinical Trial, SSI: Surgical Site Infection.

Table S6. NHLBI Quality Assessment of Controlled Intervention Studies.

NHLBI Quality Assessment of Controlled Intervention Studies																
**First Author et al., Year**	**Q1**	**Q2**	**Q3**	**Q4**	**Q5**	**Q6**	**Q7**	**Q8**	**Q9**	**Q10**	**Q11**	**Q12**	**Q13**	**Q14**	**Total Score**	**Quality Rating**
Etemadi et al., 2022	Y	Y	Y	Y	Y	N	Y	Y	Y	Y	Y	N	Y	Y	12/14 (85.71%)	Good
Dragovic et al., 2020	Y	Y	N	N	N	N	Y	Y	Y	Y	Y	N	N	Y	8/14 (57.14%)	Fair
Banche et al., 2007	Y	Y	N	N	N	Y	Y	Y	Y	Y	Y	N	Y	Y	10/14 (71.42%)	Fair
Dragovic et al., 2018	Y	Y	N	N	N	Y	Y	Y	Y	Y	Y	N	Y	Y	10/14 (71.42%)	Fair
Sala-Perez et al., 2016	Y	Y	N	Y	Y	Y	Y	Y	Y	Y	Y	N	N	Y	11/14 (78.57%)	Good
Balakrishna et al., 2022	Y	Y	Y	Y	N	Y	Y	Y	Y	Y	Y	N	N	Y	11/14 (78.57%)	Good
Oladega et al., 2019	Y	Y	Y	Y	N	Y	Y	Y	Y	Y	Y	N	Y	Y	12/14 (85.71%)	Good
Bucci et al., 2017	Y	Y	N	N	N	Y	Y	Y	Y	Y	Y	N	N	Y	9/14 (64.28%)	Fair
Gazivoda et al., 2015	Y	Y	N	N	N	Y	Y	Y	Y	Y	Y	N	N	Y	9/14 (64.28%)	Fair
Thoniyottupurayil et al., 2022	Y	Y	Y	N	N	Y	Y	Y	Y	Y	Y	N	N	Y	10/14 (71.42%)	Fair
Pelia et al., 2021	Y	Y	N	N	N	Y	Y	Y	Y	Y	Y	N	N	Y	9/14 (64.28%)	Fair
Joshi et al., 2011	Y	Y	N	N	N	Y	Y	Y	Y	Y	Y	N	N	Y	9/14 (64.28%)	Fair
El-rewainy et al., 2015	Y	Y	N	N	N	Y	Y	Y	Y	Y	Y	N	Y	Y	10/14 (71.42%)	Fair

Ghoreishian et al., 2009	Y	Y	N	N	N	Y	Y	Y	Y	Y	Y	N	Y	Y	10/14 (71.42%)	Fair

Q1: Was the study described as randomized, a randomized trial, a randomized clinical trial, or an RCT?, Q2: Was the method of randomization adequate (i.e., use of randomly generated assignment)?, Q3: Was the treatment allocation concealed (so that assignments could not be predicted)?, Q4: Were study participants and providers blinded to treatment group assignment?, Q5: Were the people assessing the outcomes blinded to the participants' group assignments?, Q6: Were the groups similar at baseline on important characteristics that could affect outcomes (e.g., demographics, risk factors, co-morbid conditions)?, Q7: Was the overall drop-out rate from the study at endpoint 20% or lower of the number allocated to treatment?, Q8: Was the differential drop-out rate (between treatment groups) at endpoint 15 percentage points or lower?, Q9: Was there high adherence to the intervention protocols for each treatment group?, Q10: Were other interventions avoided or similar in the groups (e.g., similar background treatments)?, Q11: Were outcomes assessed using valid and reliable measures, implemented consistently across all study participants?, Q12: Did the authors report that the sample size was sufficiently large to be able to detect a difference in the main outcome between groups with at least 80% power?, Q13: Were outcomes reported or subgroups analyzed prespecified (i.e., identified before analyses were conducted)?, Q14: Were all randomized participants analyzed in the group to which they were originally assigned, i.e., did they use an intention-to-treat analysis?; Total Score: Number of yes; CD: cannot be determined; NA: not applicable; NR: not reported; N: no; Y: yes. Quality Rating: Poor <50%, Fair 50–75%, Good ≥75%.

Table S7. NHLBI Quality Assessment Tool for Observational Cohort and Cross-Sectional Studies.

NHLBI Quality Assessment Tool for Observational Cohort and Cross-Sectional Studies																
**First Author et al., Year**	**Q1**	**Q2**	**Q3**	**Q4**	**Q5**	**Q6**	**Q7**	**Q8**	**Q9**	**Q10**	**Q11**	**Q12**	**Q13**	**Q14**	**Total Score**	**Quality Rating**
Parrini et al., 2023	Y	Y	N	Y	N	Y	Y	N	Y	Y	Y	N	Y	N	9/14 (64.28%)	Fair
Yaman et al., 2022	Y	Y	N	N	Y	Y	N	N	Y	Y	Y	N	N	N	7/14 (50%)	Fair
Otten et al., 2005	Y	Y	Y	N	Y	N	Y	N	Y	Y	Y	N	Y	N	9/14 (64.28%)	Fair

Q1: Was the research question or objective in this paper clearly stated?, Q2: Was the study population clearly specified and defined?, Q3: Was the participation rate of eligible persons at least 50%?, Q4: Were all the subjects selected or recruited from the same or similar populations (including the same time period)? Were inclusion and exclusion criteria for being in the study prespecified and applied uniformly to all participants?, Q5: Was a sample size justification, power description, or variance and effect estimates provided?, Q6: For the analyses in this paper, were the exposure(s) of interest measured prior to the outcome(s) being measured?, Q7: Was the timeframe sufficient so that one could reasonably expect to see an association between exposure and outcome if it existed?, Q8: For exposures that can vary in amount or level, did the study examine different levels of the exposure as related to the outcome (e.g., categories of exposure, or exposure measured as continuous variable)?, Q9: Were the exposure measures (independent variables) clearly defined, valid, reliable, and implemented consistently across all study participants?, Q10: Was the exposure(s) assessed more than once over time?, Q11: Were the outcome measures (dependent variables) clearly defined, valid, reliable, and implemented consistently across all study participants?, Q12: Were the outcome assessors blinded to the exposure status of participants?, Q13: Was loss to follow-up after baseline 20% or less?, Q14: Were key potential confounding variables measured and adjusted statistically for their impact on the relationship between exposure(s) and outcome(s)?; Total Score: Number of yes; CD: cannot be determined; NA: not applicable; NR: not reported; N: no; Y: yes. Quality Rating: Poor <50%, Fair 50–75%, Good

Referenc

1. Santos A.J.F., Monteiro J.L.G.C., Moraes S.L.D., Vasconcelos B.C.E., Pellizzer E.P. Clinical comparison of conventional suture and tissue adhesive in third molar surgeries: a systematic review. *Gen Dent.* **2023**, *75*, 25–29.
2. Stran-Lo Giudice A.F., Ortiz A.M., Sánchez-Labrador L., Cortés-Bretón Brinkmann J., Cobo-Vázquez C.M., Meniz-García C. Current status of split-mouth controlled clinical trials comparing cyanoacrylate vs. conventional suture after lower third molar surgeries: a systematic literature review. *Acta Odontol Scand.* **2023,** *81*, 349–357.
3. Petronis Ž., Zigmantavičius J., Gervickas A. Various wound closure ways after impacted lower wisdom teeth removal: A review. *Stomatologija.* **2020**, *22*, 107–115.
4. Ma S., Li X., Zhang A., Liu S., Zhao H., Zhao H. Efficacy of secondary closure technique after extraction of third molars: a meta-analysis. *Br J Oral Maxillofac Surg.* **2019**, *57*, 977–984.
5. Bailey E., Kashbour W., Shah N., Worthington H.V., Renton T.F., Coulthard P. Surgical techniques for the removal of mandibular wisdom teeth. *Cochrane Database Syst Rev.* **2020**, *7*, CD004345.
6. Azab M., Ibrahim S., Li A., Khosravirad A., Carrasco-Labra A., Zeng L., Brignardello-Petersen R. Efficacy of secondary vs primary closure techniques for the prevention of postoperative complications after impacted mandibular third molar extractions: A systematic review update and meta-analysis. *J Am Dent Assoc.* **2022**, *153*, 943–956.
7. Zhang Y., Chen X., Zhou Z., Hao Y., Li H., Cheng Y., Ren X., Wang X. Effects of Impacted Lower Third Molar Extraction on Periodontal Tissue of the Adjacent Second Molar. *Ther Clin Risk Manag.* **2021**, *17*, 235–247.
8. Faris A., Khalid L., Hashim M., Yaghi S., Magde T., Bouresly W., Hamdoon Z., Uthman A.T., Marei H., Al-Rawi N. Characteristics of Suture Materials Used in Oral Surgery: Systematic Review. *Int Dent J.* **2022**, *72*, 278–287.
9. Raut V.D., Kumar S., Raut S., Bhate K., Singh M., Kakodkar P., Waknis, P. Dehiscence rate in wound closed with cyanoacrylate and black braided silk after surgical removal of impacted third molar: A systematic review and meta-analysis. *Oral Surg.* **2022**, *15*, 17–23.
10. Borie E., Rosas E., Kuramochi G., Etcheberry S., Olate S., Weber B. Oral Applications of Cyanoacrylate Adhesives: A Literature Review. *BioMed Research International*. **2019,** *2019*, 8217602.
11. Etemadi Sh M., Rahgozar S., Tajmiri G., Alizargar J., Wu S.V. Microbiological Evaluation of the Antibacterial Vicryl Suture in the Mandibular Third Molar Surgery. *Journal of Orofacial Sciences.* **2023**, *14*, 120–127.
12. Dragovic M., Pejovic M., Stepic J., Colic S., Dozic B., Dragovic S., Lazarevic M., Nikolic N., Milasin J., Milicic B. Comparison of four different suture materials in respect to oral wound healing, microbial colonization, tissue reaction and clinical features-randomized clinical study. *Clin Oral Investig.* **2020,** *24*, 1527–1541.
13. Banche G., Roana J., Mandras N., Amasio M., Gallesio C., Allizond V., Angeretti A., Tullio V., Cuffini A.M. Microbial adherence on various intraoral suture materials in patients undergoing dental surgery. *J Oral Maxillofac Surg.* **2007**, *65*, 1503–1507.
14. Dragović M., Pejovic M., Stepic J., Dragovic S., Nikolic N., Kuzmanovic Pficer J., Colic S., Milasin J. Microbial adherence affinity and clinical characteristics of polypropylene versus silk sutures in oral surgery. *Srpski arhiv za celokupno lekarstvo.* **2018,** *146*, 258–263.
15. Sala-Pérez S., López-Ramírez M., Quinteros-Borgarello M., Valmaseda-Castellón E., Gay-Escoda C. Antibacterial suture vs silk for the surgical removal of impacted lower third molars. A randomized clinical study. *Med Oral Patol Oral Cir Bucal.* **2016**, *21*, e95–e102.
16. Balakrishna R., Poojary D.R.A., Sali S., Moharana A.K., Deepak T.S. Single-blind, randomized study comparing clinical equivalence of Trusilk ® and Mersilk ® silk sutures for mucosal closure following surgical removal of mesioangular impacted mandibular third molar. *F1000Res.* **2022**, 11, 689.

17. Oladega A.A., James O., Adeyemo W.L. Cyanoacrylate tissue adhesive or silk suture for closure of surgical wound following removal of an impacted mandibular third molar: A randomized controlled study. *J Craniomaxillofac Surg.* **2019**, *47*, 93–98.
18. Bucci M., Borgonovo A., Bianchi A., Zanellato A., Re D. Microbiological analysis of bacterial plaque on three different threads in oral surgery. *Minerva Stomatol.* **2017,** *66*, 28–34.
19. Gazivoda D., Pelemiš D., Vujašković G. A clinical study on the influence of suturing material on oral wound healing. *Vojnosanit Pregl.* **2015**, *72*, 765–769.
20. Thoniyottupurayil N., Rao H.T., Sequeira J. Tissue Adhesive or Suture for Wound Closure Following Surgical Removal of an Impacted Mandibular Third Molar: A Randomized Comparative Study. *World J Dent.* **2022**, *13*, 587–593.
21. Pelia A.K., Kaur T., Kapila S., Dhawan A., Singh Bhullar R. Efficacy of Isoamyl 2-Cyanoacrylate Tissue Adhesive and 3-0 Vicryl Rapide Sutures in Impacted Mandibular Third Molar Surgery: A Comparative Study. *AMEI's Curr Trends Diagn Treat***. 2021**, *5*, 80–84.
22. Joshi A.D., Saluja H., Mahindra U., Halli R. A comparative study: efficacy of tissue glue and sutures after impacted mandibular third molar removal. *J Maxillofac Oral Surg.* **2011**, *10*, 310–315.
23. El-rewainy M.A., Osman S.M., Hassan N.E. The use of n-butyl cyanoacrylate adhesive in the closure of mucoperiosteal flap after the surgical extraction of impacted mandibular third molar. *Medicine.* **2015**, *2015*, 165428.
24. Ghoreishian M., Gheisari R., Fayazi M. Tissue adhesive and suturing for closure of the surgical wound after removal of impacted mandibular third molars: a comparative study. *Oral Surg Oral Med Oral Pathol Oral Radiol Endod.* **2009**, *108*, e14–e16.
25. Parrini S., Bovicelli A., Chisci G. Microbiological Retention on PTFE versus Silk Suture: A Quantitative Pilot Study in Third Molar Surgery. *Antibiotics (Basel).* **2023,** *12*, 562.
26. Yaman D., Paksoy T., Ustaoğlu G., Demirci M. Evaluation of Bacterial Colonization and Clinical Properties of Different Suture Materials in Dentoalveolar Surgery. *J Oral Maxillofac Surg.* **2022**, *80*, 313–326.
27. Otten J.E., Wiedmann-Al-Ahmad M., Jahnke H., Pelz K. Bacterial colonization on different suture materials--a potential risk for intraoral dentoalveolar surgery. *J Biomed Mater Res B Appl Biomater.* **2005**, *74*, 627–635.

Disclaimer/Publisher's Note: The statements, opinions and data contained in all publications are solely those of the individual author(s) and contributor(s) and not of MDPI and/or the editor(s). MDPI and/or the editor(s) disclaim responsibility for any injury to people or property resulting from any ideas, methods, instructions or products referred to in the content.
